# A Microfluidic Platform to design crosslinked Hyaluronic Acid Nanoparticles (cHANPs) for enhanced MRI

**DOI:** 10.1038/srep37906

**Published:** 2016-11-30

**Authors:** Maria Russo, Paolo Bevilacqua, Paolo Antonio Netti, Enza Torino

**Affiliations:** 1 Istituto Italiano di Tecnologia, IIT - Center for Advanced Biomaterials for Health Care, CABHC@CRIB, Largo Barsanti e Matteucci, 80125, Naples, Italy; 2 University of Naples Federico II, Department of Chemical Engineering, Materials and Industrial Production, P.le Tecchio 80, 80125, Naples, Italy; 3 IRCCS Fondazione SDN, Istituto di Ricerca Diagnostica e Nucleare, 80143 Naples, Italy; 4 University of Naples Federico II, Department of Chemical Engineering, Materials and Industrial Production, P.le Tecchio 80, 80125, Naples, Italy

## Abstract

Recent advancements in imaging diagnostics have focused on the use of nanostructures that entrap Magnetic Resonance Imaging (MRI) Contrast Agents (CAs), without the need to chemically modify the clinically approved compounds. Nevertheless, the exploitation of microfluidic platforms for their controlled and continuous production is still missing. Here, a microfluidic platform is used to synthesize crosslinked Hyaluronic Acid NanoParticles (cHANPs) in which a clinically relevant MRI-CAs, gadolinium diethylenetriamine penta-acetic acid (Gd-DTPA), is entrapped. This microfluidic process facilitates a high degree of control over particle synthesis, enabling the production of monodisperse particles as small as 35 nm. Furthermore, the interference of Gd-DTPA during polymer precipitation is overcome by finely tuning process parameters and leveraging the use of hydrophilic-lipophilic balance (HLB) of surfactants and pH conditions. For both production strategies proposed to design Gd-loaded cHANPs, a boosting of the relaxation rate T_1_ is observed since a T_1_ of 1562 is achieved with a 10 μM of Gd-loaded cHANPs while a similar value is reached with 100 μM of the relevant clinical Gd-DTPA in solution. The advanced microfluidic platform to synthesize intravascularly-injectable and completely biocompatible hydrogel nanoparticles entrapping clinically approved CAs enables the implementation of straightforward and scalable strategies in diagnostics and therapy applications.

The Magnetic Resonance Imaging (MRI) represents the first-line diagnostic imaging modality for numerous indications. It is a clinically well-established, non-invasive technique that leverages the magnetic properties of water protons present in the body to produce three-dimensional whole body anatomical and functional images^1^,^2^.

High magnetic fields (1.5 T and above) are clinically favoured because of their higher signal-to-noise ratio, capability for MR spectroscopy^3^ and other forms of functional MRI, such as high-speed imaging and high-resolution imaging. MRI signal intensity is related to the relaxation rate of *in vivo* water protons and can be enhanced by the administration of a contrast agent (CA) prior to scanning. These CAs utilize paramagnetic metal ions to enhance the contrast in an MR image by positively influencing the relaxation rates of water protons in the immediate surroundings of the tissue in which they localize. The ability of CAs to effectively enhance image contrast depends on their relaxivity (longitudinal *r*_*1*_; transversal *r*_*2*_) and the level of accumulation at the target site^4^.

Among different CAs, Gadolinium-based ones, used in up to 30% of clinical MRI scans^5^, consist of poly-amino carboxylate complexes of Gd ions, where Gd ions cytotoxicity is sequestered via chelation with ligands such as diethylenetriaminepentaacetic acid (DTPA) and tetraazacyclododecane-1,4,7,10-tetraacetic acid (DOTA)^6^,^7^,^8^. However, despite its certain role, Gadolinium-based CAs, like most of other clinically relevant CAs, suffer from poor sensitivity^6^ and rapid renal clearance, requiring long scan times, thus severely limiting the time window for MRI. In addition, they present low tissue specificity, leading to concerns in linking the use of these CAs with nephrogenic systemic fibrosis (NSF)^9^ and progressive accumulation in various central nervous system (CNS) structures following repeated gadolinium administration^10^.

To date, several efforts have increased tissue specificity, thus reducing nephrotoxicity, by designing architectures that entrap or conjugate CAs^11^,^12^,^13^,^14^,^15^,^16^,^17^,^18^,^19^,^20^. Recently, approaches using batch conventional synthesis were shown to successfully boost relaxivity by entrapping Gd-based CAs into biocompatible matrices, without chemically modifying the clinically approved Gd-chelates^21^,^22^,^23^. Decuzzi *et al*.^4^ optimized the performance of MRI nanoconstructs by confining Gd-DTPA within porous structures of silicon microparticles (about 1–2 μm) produced by microfabrication. They examined the consequences of geometrical confinement of CAs within the nanopores, achieving a 2–3 times enhancement in r_1_ without chemical modification of the chelate; however, a considerable increase of the relaxometric properties was observed only when nanotubes or fullerenes were also incorporated into the silicon structure. Courant *et al*. enhanced relaxivity by encapsulating Gd-chelates (Gd-DOTA) into hydrophilic and biocompatible polymer nanoparticles (about 250 nm) obtained by the ionotropic gelation between Chitosan (CH) and Hyaluronic Acid (HA)^24^.

Recently, we have exploited the impact of crosslinked and un-crosslinked biopolymer matrices on relaxometric properties of CAs where the contribution to the enhancement of CAs is highlighted and attributed to the reduced mobility of water within the hydrogel^25^. However, systems with nanometric and monodisperse size under 100 nm are likely to improve delivery functions^26^ to the tissues, stability of the metal chelates, and also provide enhanced relaxometric properties of the Gadolinium-based CAs.

Nanoprecipitation represents a very promising and powerful approach to produce biopolymeric nanoparticles in batch processes^27^ even if polydispersity due to aggregation effects challenges in the complete removal of the solvents, in particular for high polymer–solvent affinities, and low loadings of hydrophilic compounds^28^. Indeed, the inherent variability and complexity of conventional batch precipitation processes often make it problematic to produce particles with well-defined physicochemical and functional attributes^27^,^29^.

To address these demands, a microfluidic platform is presenting as an ideal method to tightly control the final particle properties, i.e. size, polydispersity, etc., due to the process’s ability to accurately control process parameters and thus enables efficient, continuous and tunable mixing^30^,^31^,^32^,^33^,^34^,^35^,^36^.

Over the past decade, microfluidics has enabled the production of a variety of enhanced nanoparticles for applications in therapy and diagnostics^37^,^38^,^39^,^40^,^41^,^42^,^43^,^44^,^45^,^46^,^47^. Recently, Karnik *et al*.^36^ synthesized PLGA-PEG nanoparticles in a microfluidic channel by rapidly mixing polymer-acetonitrile solutions and water using hydrodynamic flow focusing, in a controlled nanoprecipitation process. Valencia *et al*.^48^ developed homogeneous lipid_Quantum Dots (QDs) NanoParticles (NPs) composed of CdSe/ZnS QDs coated with a lecithin and DSPE-PEG layer. Capretto *et al*.^49^ investigated the production of polymer micelles (PMs) using Pluronic tri-block copolymer in a series of microfluidic-based reactors with a hydrodynamic flow focusing configuration. Their results showed that the PM’s size was determined by the flow rate ratio, the device geometry, viscosity and fluidic conditions. Souza Bicudo *et al*.^50^ described a process for the production of Hyaluronic Acid (HA) crosslinked nanoparticles by nanoprecipitation at the interface of organic solvent/water phases. HA nanoparticles were crosslinked with adipic dihydrazide (ADH) and chloride carbodiimide (EDCL) and presented sizes ranging from 140 to 460 nm, depending on the operating conditions. However, although great efforts have been made to apply microfluidic technologies to the production of nanoparticles, the exploitation of microfluidic approaches to support the design of nanostructures to improve relaxometric properties of clinically approved CAs for MRI has not been developed.

Here, the presented microfluidic platform takes advantage of interferences caused by the presence of Gd-DTPA in flow-focused nanoprecipitation, enabling the formation of monodisperse crosslinked Hyaluronic Acid Nanoparticles (cHANPs) under 100 nm, which are able to impact relaxation rates of Gd-DTPA. Through microfluidics, we aim to achieve a fine-tuning of the mixing process among all species and thus, tight control of their nanoprecipitation behaviour, crosslinking reaction and the relaxometric properties. The proposed approach has the goal to introduce microfluidic strategies to modulate the nanoprecipitation playing on the hydrophilic-lipophilic properties of the surfactants or the pH solution conditions to optimize relaxometric performances.

## Results

### Successful conditions to produce cHANPs for MRI through a Microfluidic Flow-Focusing platform

In flow-focused nanoprecipitation^40^,^49^,^51^, the non-solvent phase, flowing through two side channels, focuses the solvent phase in the main channel inducing the mutual diffusion of all solvents and promoting the precipitation of the solute and, therefore, the production of nanoparticle morphologies.

**Figure 1:**
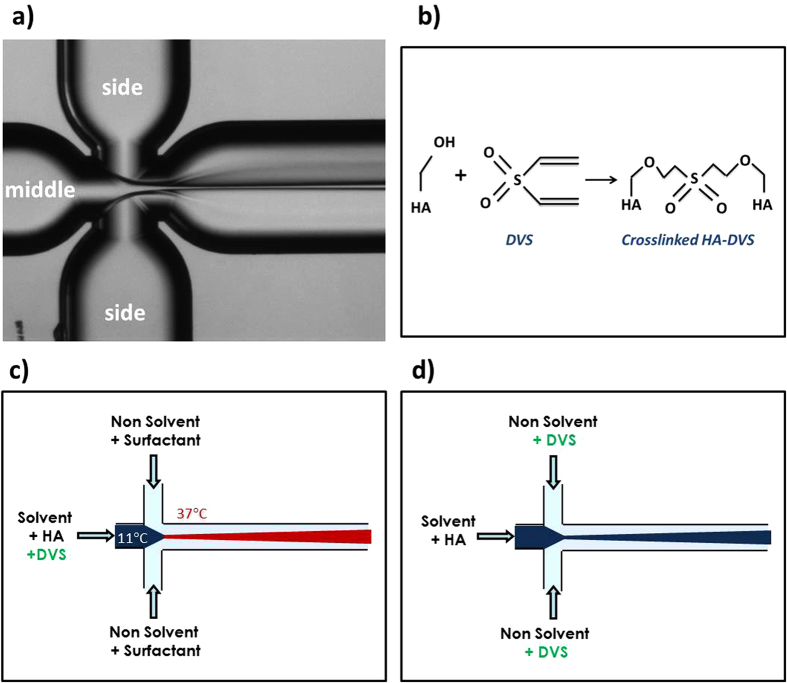
Schematic illustration of Microfluidic experimental set-up. (**a**) Optical Fluorescence Microscopy Image of Flow-Focusing pattern; (**b**) Crosslinking reaction of HA hydroxyl groups with divinyl sulfone (DVS). Qualitative Illustration of two different crosslinking strategies processed in our microfluidic device: (**c**) when DVS is added into the middle channel; (**d**) when DVS is added into the side channels.

In the synthesis of cHANPs, the solvent phase consists of a hydrogel aqueous solution while the non-solvent phase is mainly represented by the organic solvents, i.e. acetone or ethanol, plus other compounds to improve or stabilize the nanoparticles (Fig. 1a and Supplementary Figure S1). Flow rate conditions play a key role and are evaluated by analyzing the z- Average and Polydispersity Index (PDI) values obtained by Dynamic Light Scattering (DLS). Results are presented in terms of the Flow Rate Ratio FR^2^ (defined as the ratio of Volume Flow Rate Solvent and Volume Flow Rate Non-Solvent), and, on the dependence of FR^2^ ranging from 0.2 to 0.6, nanoparticle size varies from 35 nm to 500 nm (for further details see Supplementary Table S1, Figure S2). High yield process parameters facilitate rapid solvent extraction that promotes fast nucleation (at the expenses of growth rate), leading to the production of almost monodisperse nanoparticles. Relatively monodisperse nanoparticles are obtained even for all FR^2^ lower than 0.4 (Supplementary Table S1). Furthermore, Hyaluronic Acid concentration (C_HA_) also affects the nanoprecipitation, and most of our experiments are conducted at a constant C_HA_, 0.05% wt/v, allowing a longer stability of the system. However, the value of FR^2^ of 0.3 (obtained at 30 μL/min -solvent flow rate – and 100 μL/min – non-solvent flow rate) and C_HA_ of 0.05% wt/v are considered as “standard flow conditions” for all the experiments (Supplementary Figure S3a and b).

We next investigate the role of HA/CA ratio on the effectiveness of microfluidic mixing observing the obtained morphologies in the absence of the crosslinking reaction (Supplementary Figure S4). A strong influence of Gd-DTPA on the focused stream and, therefore, on the precipitation is observed, which induces a significant increase in the nanoparticle’s size and their uncontrolled shape. Further investigations are required to address this behaviour towards the control of the hydrogel nanoparticles (Supplementary Figure S5). Therefore, the formulation of rational crosslinking strategies is needed to control the nanoprecipitation and stability of cHANPs in the presence of Gd-DTPA. In this perspective, Divinyl Sulfone (DVS) has been selected among different highly reactive crosslinking agents for HA. Indeed, the biocompatibility of the HA-DVS, i.e. sulfonyl bis-ethyl linkages between the hydroxyl groups of the polymer chains (Fig. 1b), has extensively been confirmed by histological analysis^52^, making our cHANPs appropriate for the potential clinical application.

### Crosslinking and Swelling behaviour of cHANPs

In the presence of Gd-DTPA, while nanoprecipitation occurs, two different crosslinking strategies are exploited to perform a simultaneous reaction: the injection of DVS into the middle channel or the side channels (Fig. 1c and d, Supplementary Figure S6). These rational strategies are discussed in term of concentration and role played by pH and HLB of selected surfactants (i.e. Span 80, Tween 21 and 85), used to avoid swelling, support the streams during the focusing action and modulate the nanoprecipitation.

**Figure 2:**
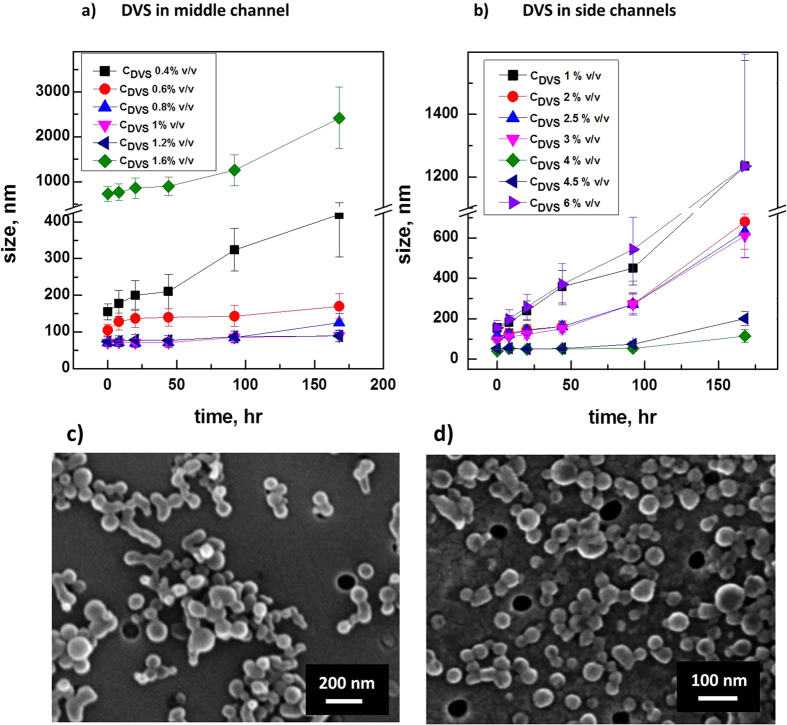
Study of the Swelling behavior. Swelling behavior regarding nanoparticles size observed at several time points for different C_DVS_ when (**a**) DVS is added in the middle channel, at standard process conditions, C_Span80_ of 0.5% v/v and 35 °C; and (**b**) DVS is added in the side channels, at pH equal to 12.3. FE-SEM images of cHANPs in water at the time zero when (**c**) 0.8% v/v of DVS is added in the middle channel; (**d**) 4% v/v of DVS is added in the side channels.

In the first approach, Temperature is also strictly controlled to avoid the onset of the crosslinking reaction in the HA aqueous solution before precipitation happens. During injection into the microfluidic device, the polymer solution within the syringe is kept constant at 5 °C while the device is heated at 35 °C, to promote the reaction at the nozzle section, where the mixing takes place. In this approach, nanoparticles as small as 70 nm are produced at DVS concentrations of 0.6–0.8% v/v at previously defined “standard flow conditions” and C_Span80_ 0.5% v/v (Fig. 2a, time point at zero seconds). A comprehensive summary of the effects of the Temperature and surfactants on the flow-focusing behaviors are reported in the Supplementary Figure S7 and Table S2.

In the second approach, smaller nanoparticles of about 40 nm are formed, at the “standard flow conditions” and pH equal to 12.3, by adding DVS up to 4% v/v to the side channels (Fig. 2b, time point at zero seconds). Several experiments are also performed by adding surfactants simultaneously with DVS to the side channels, but in this last case, both the morphologies and the crosslinking reaction result entirely compromised. Both our chosen crosslinking approaches have the advantage to be highly tunable through process parameters and temperature. In our system, the competition between crosslinking reaction and water extraction could mainly affect the second strategy, where an additional barrier to the reaction is represented by DVS diffusion from the side flows to the main one.

Differences in the crosslinking degree of the nanoparticles are evaluated by the swelling behaviour under physiological conditions for both proposed strategies. In the first strategy, a lower amount of crosslinker is used to reduce the swelling behaviour even after several hours. Indeed, stable nanoparticles are obtained for C_DVS_ ranging from 0.8 to 1.2% v/v. The increase of C_DVS_ at more than 1.2% v/v, however, promotes an instability of the flow or compromises the crosslinking reaction and, consequently, the swelling (Fig. 2a and c). Compared to the first approach, for the other one, a higher amount of agent is added to avoid a rapid swelling behaviour of nanoparticles in water. In particular, for C_DVS_ ranging from 4 to 4.5% v/v, the swelling behaviour of the cHANPs is not observed even after several days. Higher or lower concentrations of DVS lead to the formation of swelling particles or flow instability (Fig. 2b and d). Once again, the comparison between the two proposed strategies highlights considerable differences in the nanoparticle behaviour. Indeed, the smaller size and the absence of swelling behaviour obtained in the first strategy can be related to a faster and efficient crosslinking reaction due to more favourable reaction conditions in the aqueous environment, an ideal pH and temperature able to promote a faster crosslinking among the polymer chains. Additional data at higher concentration of DVS, resulting in agglomeration and undefined morphologies or absence of nanoparticle formation, are not reported.

### Loading Capability, Encapsulation Efficiency and Surface properties of Gd-loaded cHANPs

The proposed strategies also have the capability to increase or control the encapsulation efficiency (EE) in the cHANPs, to prevent the waste of expensive compounds and sustain their dosage over an extended treatment period. Results show that a DVS concentration added to the middle or the side phase has a significant impact on the entrapment efficiency of cHANPs. In details, for DVS in the middle channel, it is observed that an increase in DVS concentration (from 0.6 to 1% v/v) results in a rise of cHANPs encapsulation efficiency from 27 to 89% and a loading capability from 22 to 59%. On the contrary, a worse control is observed by increasing the concentration of DVS in the non-solvent phase, where a slight increase in cHANPs encapsulation efficiency and loading capability is obtained from 39 to 53% and from 26 to 35%, respectively, for selected DVS values of 4 and 4.5% v/v. Loading capability (LC) is only reported for stable nanoparticles in water and it is also compared with loading capability resulting by the encapsulation of a highly toxic and non-chelate Gadolinium, GdCl_3_ at equal conditions, proving that values reached are of about 90% (see the specific paragraph in Supplementary Information). The importance of a high encapsulation efficiency has been already emphasized since a large nanoparticles recovery is required for reducing manufacturing costs while size and morphology are important for quality control and biodistribution of injectable products. Furthermore, surface properties analysis of the empty cHANPs or cHANPs containing Gd-DTPA is conducted to assess the promising delivery functions of the cHANPs. Gd-free cHANPs have a negative surface charge of −50 mV (±4.5 mV) which can be attributed to the presence of carboxylic end groups of the polymer on the nanoparticle surface, proposing their availability to future decorations of the nanoparticles. Zeta Potential measurements show a significant increase to −36.4 mV (±3.01) linked to the encapsulation of Gd-DTPA^26^.

### *In vitro* MRI

**Figure 3:**
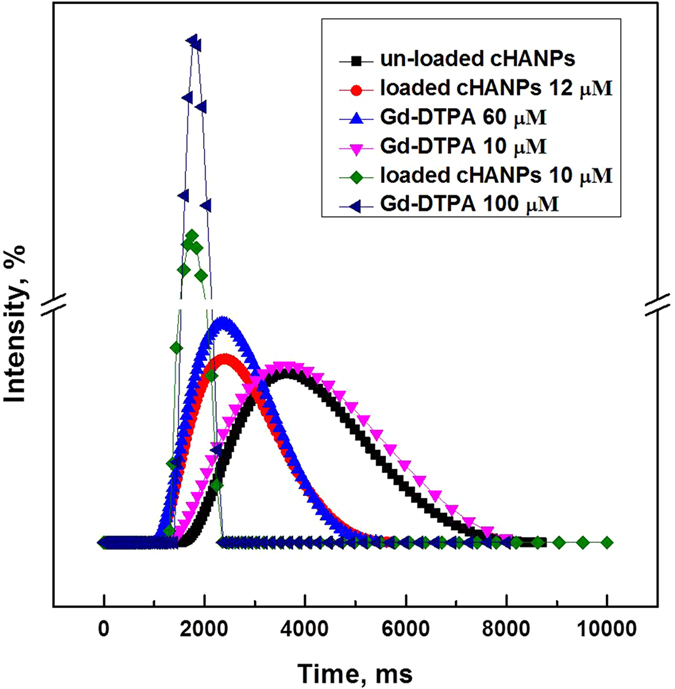
*In vitro* relaxation time distribution. Relaxation time distribution reported for: Gd-DTPA in water solution at (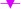) 10 μM, (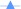) 60 μM and (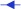) 100 μM; un-loaded cHANPs (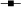); loaded cHANPs at *standard conditions* obtained using (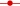) 4% v/v DVS in the side channels, at pH 12.3, reported at 12 μM of Gd-DTPA, (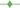) 0.8% v/v DVS and C_span80_ 0.5% v/v in the middle channel, reported at Gd-DTPA of 10 μM.

The relaxation time values of Gd-DTPA and cHANPs are calculated at 37 °C and 1.5 T. A notable change in relaxation rate is found for C_DVS_ at 4% v/v in the side channels and at 0.8% v/v in the middle channel. Comparison between the relaxation time distributions of loaded cHANPs, at the same concentration of Gd-DTPA in HA solution, is reported in Fig. 3. Results clearly show that the relaxation time for Gd-DTPA entrapped within cHANPs is shorter than that of the “free” contrast agent. By tuning the process parameters and adjusting the crosslinking reaction, a T_1_ of 1562 ms is achieved with 10 μM of Gd-loaded cHANPs suspension, while 100 μM of Gd-DTPA free in solution is required to achieve a comparable T_1_. Indeed, the relaxation time reported for cHANPs is achieved with a concentration about 10 times lower than that of the free Gd-DTPA. The value related to the unloaded cHANPs is also reported, proving that the nanoparticles do not contribute themselves to the relaxivity (Fig. 3).

As well-known^53^, the relaxivity is defined as the rate of change in relaxation times of the water protons per mM concentration of metal ions. Larger is the relaxivity of a given CA and larger is the induced spin-lattice relaxation time T_1_ and spin-spin relaxation time T_2_ shortening. In the presence of CAs, T_1_ and T_2_ may be shortened considerably. However, an increase in contrast agent concentration provides an increase in signal intensity due to the effect on T_1_ until a certain optimal concentration is reached while further increase in concentration reduces the signal because of a broad effect on T_2_, producing as net result a non-linear relation. This peculiar behaviour dictates their use in clinical practice, preferring contrast agents that have a relatively greater effect on T_1_ than on T_2_ and reveals the enormous advantage of manipulating clinical relevant contrast agents with enhanced MRI sensitivity. The relaxation times of the selected cHANPs samples remain unchanged at standard physiological conditions for at least 5 days after sample preparation, ensuring no leakage of Gd-DTPA from the cHANPs during MRI acquisitions.

## Discussion

To date, Gadolinium chelates are the most used in the clinical MRI. Every year, more than 10 million of MRI scans are performed with CAs, because of their relatively high stability and inertness in the body even if they still suffer from tissue specificity and exhibit low relaxivity^54^. Several strategies have been investigated to develop better CAs with high relaxivity, low toxicity, and tumor specificity through the design and development of several classes of Gd-based MRI contrast agents (CAs) for tumor imaging. However, the exploitation of microfluidics to produce hydrogel nanostructures with increased MR performances is still missing.

In this work, we have coupled a flow focused nanoprecipitation to an efficient crosslinking reaction based on Divinyl Sulfone (DVS) to entrap the relevant clinical Gd-DTPA in crosslinked Hyaluronic Acid Nanoparticles (cHANPs) able to increase its relaxometric properties without the chemical modification of the chelate (Fig. 1).

Typically the microfluidics enables a fast mixing in the microchannels promoting the formation of nanomaterials at laminar flow condition^51^. In our system, we have individuated a threshold value FR^2^ of 0.3 below which it is possible to obtain more monodisperse and stable nanoparticles as low as 100 nm than the ones produced by similar bulk and microfluidic approaches^55^ (Supplementary Figures S1–S3). Furthermore, this threshold value is valid for high molecular weight polymers and at several polymer concentrations and different solvent/non-solvent couples. Our Microfluidic Flow-Focused nanoprecipitation approach has the potential advantage of being able to generate a wider range of slot sizes of hydrogel structure while maintaining monodispersity compared to similar bulk systems^27^. Furthermore, the flexibility of the microfluidic process allows the tuning of the nanoparticles’ size for a specific disease through changes of the hydrodynamic boundary conditions.

When Gd-DTPA is added to the polymer solution, a significant flow instability is suddenly detected even at a low concentration while no effect is detected even at high GdCl_3_ concentration (Supplementary Figure S4). We believe that the Gadolinium complex acts probably as a salt compound, increasing the hydrogel strength significantly. A possible explanation can be that the electrostatic repulsion resulting from the charged groups on polymer chains is suppressed by the accumulation of counterions due to the metal complex^56^. Furthermore, starting from the considerations reported by Gouin *et al*.^57^ about the interaction between DTPA and HA, we hypothesize that Gd-chelate modifies the affinity of the polymer solution shifting the supersaturation to a low degree and leads to a slow heterogeneous nucleation followed by the growth of produced nuclei into large or aggregated particles. These specific interactions induce flow perturbation, causing an uncontrolled size variation and formation of aggregated morphologies. A qualitative evaluation of nucleation and growth by diffusion is extensively discussed, and related images are reported in the Supplementary Figure S8a–c, where a comparison between the traditional^16^ and microfluidic nanoprecipitation has been carried out to interpret the Gd-DTPA interference and to explain the design of the rational strategies that could systematically ensure the entrapment of Gd-DTPA and the control of its relaxometric properties.

In particular, for the first strategy, the optimization of the flow-focusing pattern is found to be strongly dependent on the hydrophobicity and hydrophilicity of the surfactants (further results are reported in the Supplementary Information). However, the main outcome of this approach is the increase of the loading capability that can be attributed to the repulsion between surfactant and water phase. Indeed, results clearly demonstrate that the retaining of the Gd-CAs is improved by the hydrophobic nature of the surfactant that increases the interface viscosity and prevents Gd-DTPA diffusion towards the external non-solvent phases. This behaviour is also explained by the higher molecular weight of the CAs, whose extraction, in these conditions, is less favoured compared to the small water molecules. These findings are of crucial importance because the use of a surfactant is very easy to enforce compared to other alternatives, such as the use of a co-solvent, reduced concentration or addition of reacting components that could interfere with the reaction yield and contaminate samples.

Later, the macrocyclic molecules have been firmly entrapped within the hydrogel matrix, using a crosslinking reaction simultaneously occurring with the nanoprecipitation (Fig. 2). Investigations related to the addition of the DVS in the middle channels or into the side channels have attributed to the hydrogel nanoparticles some peculiar properties responsible for the modulation of the release behaviour and swelling properties.

*In vitro* MRI results (Fig. 3) proved that using our flexible platform it is possible to take advantages from the strong interference detected by the presence of Gd-DTPA producing Gd-entrapped nanoparticles with enhanced MRI properties. This observation is crucial to lead potentially to a significant reduction of administration dosage on clinical usage of T_1_ contrast agents and to gain advantages in the imaging modalities based on nanotechnologies. Indeed, the nanoparticles are widely used for the improvement of imaging techniques and all the tunability features reported for our system can potentially reduce limitation linked to a fast clearance from the bloodstream and low detection due to the dependence on the concentration^2^.

The proposed approaches aim to overcome some drawbacks of the traditional procedures for the production of nanoparticles such as high polydispersity, expensive and time-consuming purification/recovery steps^58^. Furthermore, results present the effective strategy to dose all species and to control properly the entrapment of CAs within the hydrogel nanostructures that influences MRI performances in the signal intensity and, potentially, the tissue specificity (Fig. 3).

## Conclusions

Crosslinked Hyaluronic Acid Nanoparticles (cHANPs) to be applied in MRI field have been developed and loaded with a clinically relevant Gd-CA, Gd-DTPA. cHANPs performances have been assessed in terms of longitudinal relaxation rates as a function of the crosslinking degree and loading conditions.

For the first time, an interference of the Gd-DTPA in nanoprecipitation mechanism is reported which has added some significant advances in the basic knowledge of the interactions between Gd chelates and hydrogel matrix.

We have demonstrated that, through our microfluidic strategies, it is possible to take advantages from the observed interference between HA and Gd-DTPA promoting an enhancement of the relaxometric properties of the Gd-DTPA loaded in cHANPs without chemically modifying the approved Gd-metal chelate. This microfluidic system is also proposed to overcome some drawbacks of the traditional procedures for the production of completely biocompatible and injectable hydrogel nanoparticles under 100 nm with well-defined physicochemical diagnostic properties. The designed strategies have allowed a fine control of the nanoparticle properties, such as the monodisperse average size of 40 nm, surface charge and the loading capability of 59%. Additionally, a relaxation rate T_1_ of 1562 ms is achieved with 10 μM of Gd-loaded cHANPs while 100 μM of Gd-DTPA solution is required to reach similar T_1_ (about 1724 ms) as showed in Fig. 3. A controlled degradation and release behaviour up to 96 hr under physiological conditions, high encapsulation efficiency of the hydrophilic metal chelate up to 89% are also reported. Furthermore, easy and full recovery of the cHANPs, without time-consuming and expensive purification steps with respect to the conventional processes, is taken into account.

Results support the concept that the entrapment of the Gd-based CAs within the hydrogel structure can enhance relaxivity, thus enable potentially low dosage administration. Therefore, the positive contrast effect is preserved, ensuring an improvement of the time window for clinical imaging acquisitions due to the reduction of long scan time and rapid renal clearance. Proposed cHANPs could improve the stability and reduce toxicity related to the Gd-chelate. Furthermore, the surface charge analysis reports the presence of carboxylic groups that can be readily functionalized with targeting moieties, increasing potentially chemical functionalities to impact on tissue specificity and integrating additional imaging and therapeutic capabilities.

## Materials and Methods

### Materials

Sodium Hyaluronate (M_w_ = 42 kDa) was purchased from Bohus Biotech (Sweden). Diethylenetriaminepentaacetic acid gadolinium(III) dihydrogen salt hydrate Gd-DTPA (M_w_ = 547.57 g/mol; Span80; Tween21; Tween85, Divinyl Sulfone (or Vinyl Sulfone) contains <650 ppm hydroquinone as inhibitor; purity 97%; density 1.117 g/ml at 25 °C (lit.); molecular formula C_4_H_6_O_2_S; M_w_ = 118.15 mp), Acetone (CHROMASOLV, for HPLC, ≥99.8%; molecular formula HC_3_COCH_3_; M_w_ = 58.08), Ethanol (ACS reagent, ≥99.5% (200 proof), absolute; molecular formula CH_3_CH_2_OH; M_w_ = 46.07, Sodium Hydroxide NaOH (ACS reagent, ≥97.0%, M_w_ = 40.00), Gadolinium Chloride Solution GdCl_3_(M_w_ = 263.61), Sodium Chloride NaCl (ACS reagent, ≥99.0%, M_w_ = 58.44) were purchased by Sigma-Aldrich Co. The water, used for synthesis and characterization, was purified by distillation, deionization, and reserve osmosis (Milli-Q Plus).

### Microfluidic set-up for flow-focusing approach

A quartz microfluidic device “X- Junction Chip, 190 μm”, purchased from Dolomite Centre Ltd, is used to perform all the experiments. The device has a flow focusing geometry with a 90° angle between the inlets to enhance the diffusion process (Figure S1a and b). The device is connected to 2.5–5 mL glass syringes (Microsyringes ILS Innovative Labor System) with FEP tubing (1/16″ × 0.25 mm) controlled by Nemesys system. Three-way isolation ETFE valves, connecting syringes with the microfluidic device, make the automatic fill-in of the syringes feasible, thus allowing a continuous dispensing of reagents. The linkage between FEP tubes and device is carried out through a specially designed connection with PTFE connectors. The flow-focusing behaviour on the microchannel is observed using an Optical Fluorescence Microscope (Olympus IX71) with a 4 or 10 x scanning objective (Fig. 1a).

### Production of nanoparticles by nanoprecipitation in microfluidics

Different flow rates were tested, and the influence of the *Flow Rate Ratio FR*^*2*^, defined as the ratio of Volume Flow Rate Solvent and Volume Flow Rate Non-Solvent, was determined. For the feasibility study, a 5 mL aqueous solution containing HA concentrations ranging from 0.01 to 0.1% wt/v was used to explore the effects of the nanoprecipitation by flow-focusing exclusively due to the concentration of the polymer. The initial solution was kept under continuous stirring for at least 4 hr and then injected through the middle channel. The flow rate of the middle channel was changed from 5 to 100 μL/min. Acetone or Ethanol, used as non-solvent and injected through the side channels, were laterally injected to induce nanoprecipitation by a non-solvent extraction. The flow rates of the side channels were ranged from 50 μL/min to 300 μL/min, increasing each step of 10 μL/min. Precipitated nanoparticles were collected in a Petri glass containing about 25 mL of non-solvent and kept under continuous stirring. Each experiment was repeated at least 10 times (see Supplementary Information).

### Study of Gd-DTPA interference on the nanoprecipitation

After selecting the best FR^2^, the influence of the Gadolinium on nanoprecipitation was also evaluated. The entrapping of GdCl_3_ or Gd–DTPA was exploited at several weight ratios HA/Gd ranging from 1:0.05 to 1:10, adding the metal complex to the HA aqueous solution. Loading capability and encapsulation efficiency were calculated by Induced Coupled Plasma (ICP-MS) - NexION 350. Nanoparticles were suspended in a solution of deionized (DI) water at a concentration of 150.000 particles/mL. All data were collected and processed using the Syngistix Nano Application Module. Gd was measured at m/z 157 using a 100 μs dwell time with no settling time.

### Preparation of crosslinked Hyaluronic Acid Nanoparticles (cHANPs)

In the literature studies, the chemical reaction between HA and DVS is performed at high pH values (0.2 M NaOH, 0.1 M NaCl, pH > 13)^59^,^60^,^61^. In our microfluidic system, a study on the reagents was conducted to create, at the nozzle section, sulfonyl bis-ethyl linkages between the hydroxyl groups of the polymer chains forming nanoparticles (Fig. 1b). Several experiments are carried out to study the effect of NaCl and NaOH to promote the reaction and to reduce suddenly growth’s step after the nucleation phase. The crosslinking agent was injected alternatively into the middle channel (Fig. 1c) or in the side channels (Fig. 1d) at different concentrations, ranging from 0 to 20% v/v and from 0 to 8% v/v, respectively. In some strategies, the device is heated at 35 °C through a heat chamber. In our system, the occurrence of the solvent extraction produces the dilution of water into the main stream, slightly increasing the reaction time. For these reasons, to promote the crosslinking reaction, purification and dialysis in water of the collected nanoparticles are performed after 4 hr of continuous stirring.

### Effect of the surfactant concentrations and pH conditions on the system parameters

All experimental conditions were tested by adding NaOH and NaCl to the HA solution at the concentrations ranging from 0.1 M to 0.3 M and 0.02 and 0.2 M, respectively.

Alternately, three surfactants were tested at different reagent concentrations for all experimented flow rates. Tween 85 (ranging from 0.5% v/v to 1% v/v), Tween 21 (ranging from 0.5% v/v to 3% v/v) and Span 80 (ranging from 0.5% v/v to 1.5% v/v) were mixed to the non-solvent or to the aqueous solution. Experiments were conducted without the addition of DVS.

### Stability and swelling behaviour of the cHANPs

Because of the clinical relevance of Gd-DTPA and, on the contrary, the high cytotoxicity of the GdCl_3_, stability studies were optimized only on Gd-DTPA. The Gd-DTPA loaded cHANPs were mixed with 150 μL of Phosphate Buffered Saline (PBS). The nanoparticles were kept under shaking at 150 rpm at 37 °C. The solution was divided into two equal parts and observed at 12, 24, 48, 96 and 172 hr post incubation. ICP-MS was used to assess a one-half of the solution for the total concentration of Gd^3+^ ions loaded within the nanoparticles. The other half of the solution was filtered using 0.45 μm filter, and the filtrate was analyzed for Gd^3+^ ions.

### *In vitro* MRI

Empty nanoparticles and nanoparticles containing different concentrations of GdCl_3_ or Gd-DTPA were tested by *in vitro* MRI and results were compared with Magnevist, Gd-DTPA and GdCl_3_ in water as a control. After vigorous stirring, changes in relaxation time (T_1_) were evaluated at 1.5 Tesla by Minispec Bench Top Relaxometer (Bruker Corporation) by adding 300 μL of the sample in a glass tube^45^. The relaxation time distribution was obtained by CONTIN Algorithm. The relaxation spectrum was normalized with respect to the CONTIN processing parameters. The integral of a peak corresponds therefore to the contribution of the species exhibiting this peculiar relaxation to the relaxation time spectrum^62^. Experiments were repeated at least five times.

## Additional Information

**How to cite this article**: Russo, M. *et al*. A Microfluidic Platform to design crosslinked Hyaluronic Acid Nanoparticles (cHANPs) for enhanced MRI. *Sci. Rep.* **6**, 37906; doi: 10.1038/srep37906 (2016).

**Publisher's note:** Springer Nature remains neutral with regard to jurisdictional claims in published maps and institutional affiliations.

## Supplementary Material

Supplementary Information
