# Supplementary material for: A Microfluidic Platform to design crosslinked Hyaluronic Acid Nanoparticles (cHANPs) for enhanced MRI
Source: Sci Rep. 2016 Nov 30;6:37906. doi: 10.1038/srep37906 (PMC5128828; doi:10.1038/srep37906)
Supplement: Supplementary Information [file srep37906-s1.pdf]

Supplementary Information

A Microfluidic Platform to design crosslinked Hyaluronic Acid Nanoparticles (cHANPs) for enhanced MRI

Maria Russo[a,b], Paolo Bevilacqua [a,c], Paolo Antonio Netti[a,b,d] and Enza Torino[a,d]*

[a] Istituto Italiano di Tecnologia, IIT - Center for Advanced Biomaterials for Health Care, CABHC@CRIB , Largo Barsanti e Matteucci, 80125, Naples, Italy

[b] University of Naples Federico II, Department of Chemical Engineering, Materials and Industrial Production, P.le Tecchio 80, 80125, Naples, Italy

[c] IRCCS Fondazione SDN, Istituto di Ricerca Diagnostica e Nucleare, 80143 Naples, Italy.

[d] University of Naples Federico II, Department of Chemical Engineering, Materials and Industrial Production, P.le Tecchio 80, 80125, Naples, Italy

*Corresponding author: enza.torino@iit.it

Microfluidic set-up

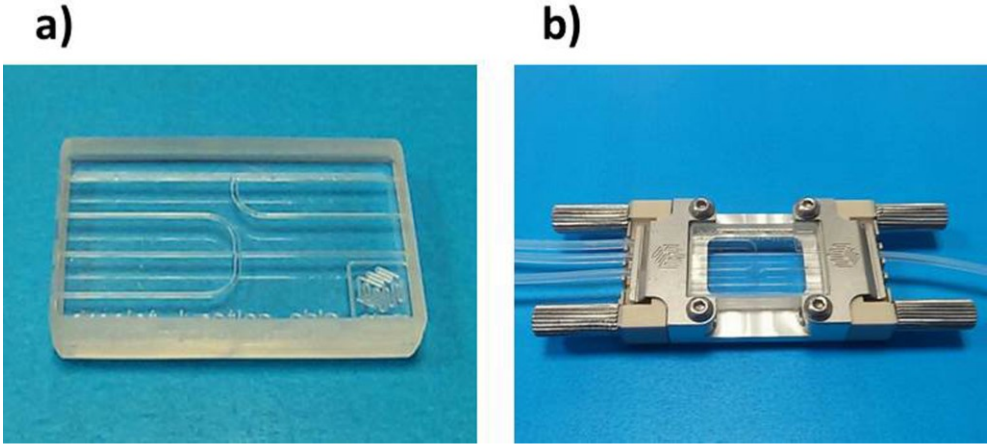

Figure S 1. Microfluidic Device. a) "Droplet Junction Chip 190 µm" with two configurations T- and X- junction, which can be used in combination. b) Droplet Junction Chip is compatible with Chip interface H for fluidic connections.

Nanoparticles purification, recovery and characterization

Purification was performed by a solvent gradient dialysis (Spectra Por Cellulose Membrane 6 –Mw cut-off MWCO 50.000) or by ultracentrifugation. A typical procedure consisted of loading collected samples into the dialysis tube and keeping the solution under continuous stirring at 130 rpm. Water was slowly added to the solution creating a concentration gradient and avoiding aggregation and diffusion phenomena across the membrane. Dynamic light scattering (DLS) was used to determine nanoparticle size and surface charge (Zeta sizer, Zeta Potential Malvern) (Table S1). In DLS analysis, the z-Average value represented the mean value of the hydrodynamic diameter of the particle, while the polydispersity index measured the width of the particle size distribution. Nanoparticles were concentrated by ultracentrifugation. The recovery was performed at 15° C, at 80000 rpm for 45 min. After these treatments, a hundred microliters of purified samples were deposited on a polycarbonate Isopore Membrane Filter (0.015, 0.05, 0.1 and 0.2 µm) by ultrafiltration vacuum system. The precipitated or deposited particles were coated with gold-palladium, and their morphology was observed by ULTRA PLUS Field Emission Scanning

Electron Microscope (FE-SEM Carl Zeiss, Germany). Transmission Electron Microscope (TEM) was also used to characterize the samples.

Influence of Flow Rate Ratio on nanoparticle morphology

Different flow rates are tested and the influence of the Flow Rate Ratio FR^2 is evaluated. Flow conditions of greater interest are selected analyzing the values of z- Average and of PDI obtained by Dynamic Light Scattering (DLS). FR^2 of interest ranges from 0.2 to 0.6 and the average diameter of the nanoparticles varies from 35 nm to 500 nm (Table S1).

In DLS measurement, the z-Average value represents the mean value of the hydrodynamic diameter of the particle, while the polydispersity index measures the width of the particle size distribution related to the standard deviation σ.

Solvent Flow Rate [μL/min]	Non Solvent Flow Rate [μL/min]	Flow Rate Ratio S/NS	z-Average [nm]	PDI	SD (±) [nm]
40	100	0.4	218.2	0.138	40.58
	80	0.5	364.6	0.130	65.83
	60	0.67	480.8	0.087	70.99
	50	0.8	523.2	0.152	101.92
	30	1.3	1001.3	0.083	144.46
30	100	0.3	35	0.264	9
	80	0.37	86.5	0.199	19.30
	60	0.5	246	0.098	38.46
	50	0.6	341.8	0.157	67.74
	40	1	684.4	0.386	212.57
	30	1.5	890.1	0.721	377.78
20	100	0.2	40	0.242	9.83
	80	0.25	53	0.172	10.99
	60	0.3	58	0.169	11.92
	50	0.4	128.9	0.144	24.46
	30	0.6	197.7	3.706	190.3
	20	1	491.5	0.598	190

Table S 1. Dynamic Light Scattering Results at different FR^2 in terms of z-Average and Standard Deviation

Through the increase of the non-solvent Flow Rate and, therefore, the reduction of FR^2, a more stable and narrow hydrodynamic focusing is achieved (Figure S2a). In this situation the extraction is faster and, therefore, nucleation is favored to the detriment of the growth, obtaining nanoparticles smaller and monodisperse. Results are confirmed by low standard deviation values (Table S1). A higher FR^2 increases the width of the middle stream, requiring a longer time to perform the mixing by diffusion and the non-solvent extraction by means of the nearby streams. This effect results in a larger amount of solvent in the final mixture possibly causing core swelling, hence the formation of large nanoparticles (Figure S2c and d).

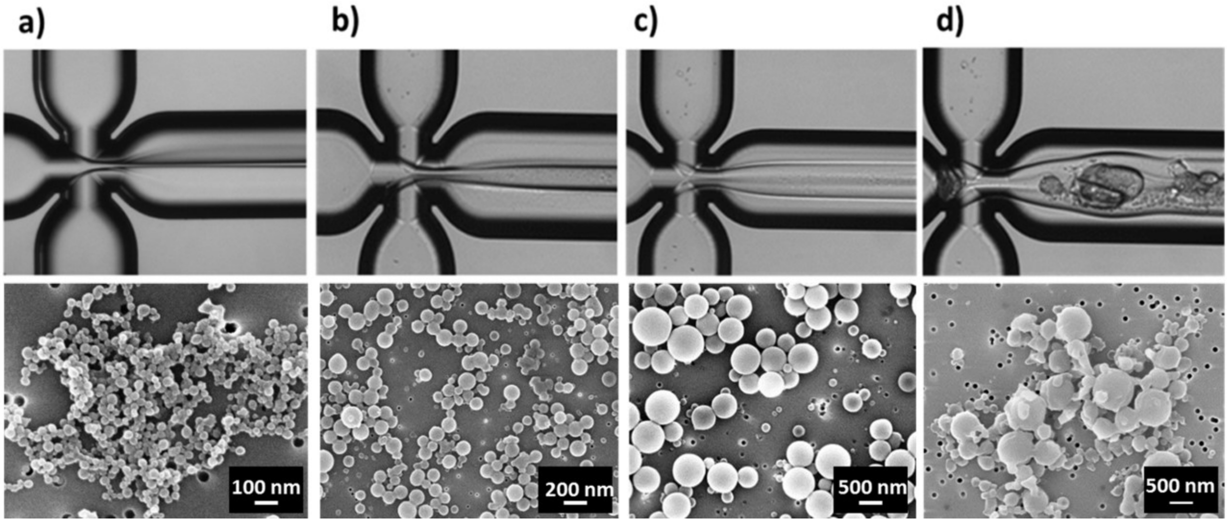

Figure S 2. Optical Fluorescence Microscope images of Flow-Focusing at several FR^2: a) 0.3; b) 0.4; c) 0.8; d) 1.5 and FE-SEM images, respectively**.**

As reported by Capretto[1], according to the mass conservation principle, the volume of sample liquid that passes through the inlet channel Q_2 must match the volume of the focused stream Q_f:

$$Q_2 = v_2 w_2 h = v_f w_f h = Q_f$$

$$w_f = \frac{Q_2}{v_f h}$$

w_f and w_2 represent the width of the focused stream and middle inlet channel, respectively. Q_2 and Q_f are the volumetric flow rates of the middle inlet channel and focused stream, respectively. h is the height of the channels, and v_2 and v_f are the average velocity of the flow in the central inlet channel and of the focused stream, respectively. Moreover, the presence of precipitate in the mixing channel and the width of hydrodynamic focusing are considered. Therefore, the selected optimal conditions provide fine focusing and absence of a precipitate in the mixing channel even for several hours. For these reasons FR^2 of 0.3 (obtained at 30 μL/min -solvent flow rate – and at 100 μL/min – non-solvent flow rate) and C_{HA} of 0.05% wt/v is considered as an optimal condition (Figure S3a and b).This choose is explained by the prolonged stability of the hydrodynamic flow-focusing, the absence of massive precipitation and a higher yield in terms of nanoparticle collection and monodispersity. Field Emission Scanning Electron Microscopy (FE-SEM) images of the collected morphologies at size vs FR^2 and size vs C_{HA} are reported in Figure S3c, d, and e.

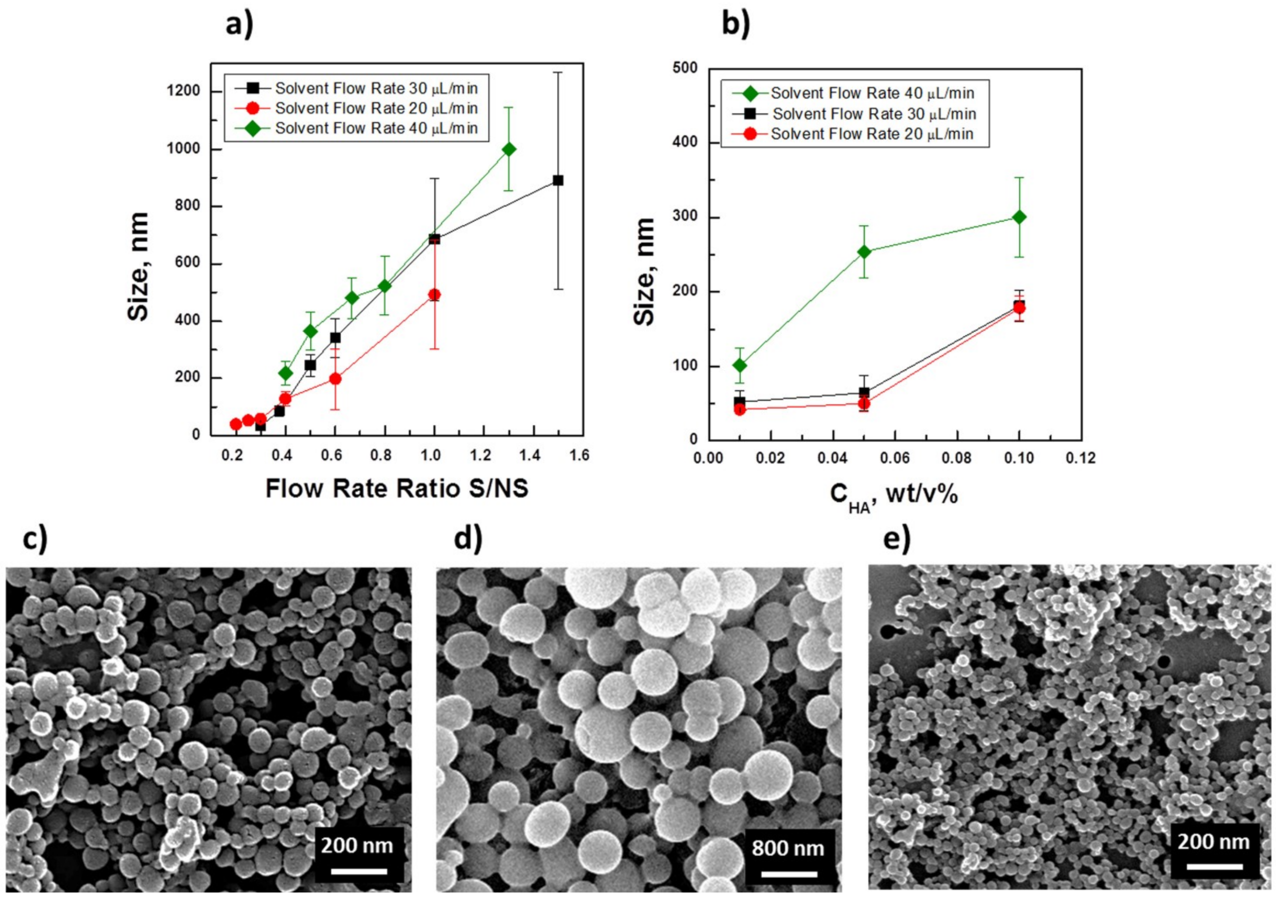

Figure S3. Feasibility Study. a) Nanoparticles Size *versus* different Flow Rate Ratio FR^2 at constant Solvent Flow Rate: 30 µL/min (- ■ -); 20 µL/min (- ● -); 40 µL/min (- ♦ -) for Hyaluronic Acid concentration C_{HA} of 0.05% wt/v. b) Nanoparticles Size *versus* C_{HA} (0.01% wt/v; 0.05% wt/v; 0.1% wt/v), at FR^2 of 0.3, obtained at Solvent Flow Rate 30 µL/min (- ■ -); 20 µL/min (- ● -) ; 40 µL/min (- ♦ -). Field Emission Scanning Electron Microscopy (FE-SEM) images of Nanoparticles at FR^2 of c) 0.4; d) 1.5; e) 0.3.

Interference of Gadolinium-based Contrast Agents on the nanoprecipitation

We next investigate the role of Gd-based CAs, $GdCl_3$ and Gd-DTPA, on the nanoprecipitation. The effect of the metal compounds is observed, and a comparison is made regarding the stability of the flow-focusing in the presence of these two CAs; furthermore, their influence on the morphology is also reported.

Figure S4 reports a change in nanoparticle size obtained, at the standard conditions, by varying the concentration of CAs (C_{CAs}), Gd-DTPA and $GdCl_3$, alternately.

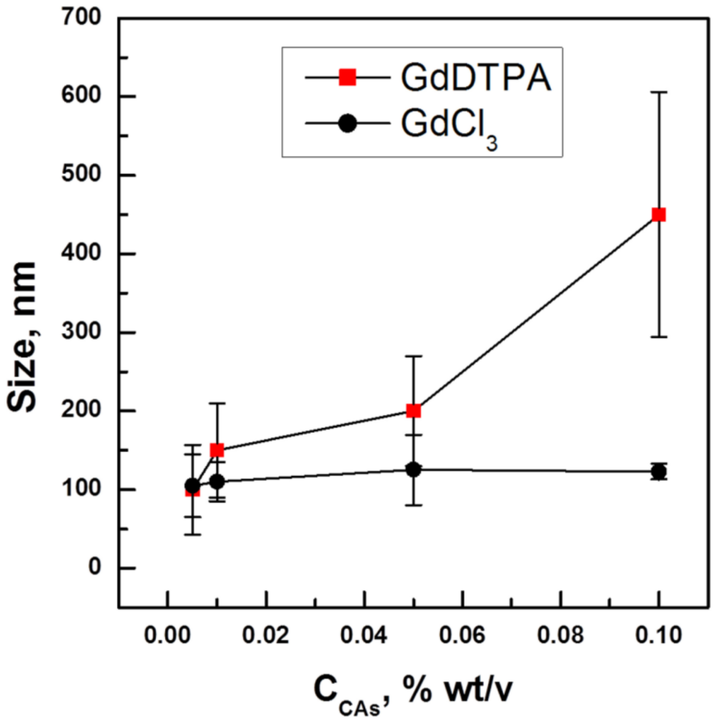

Figure S4. Influence of Gadolinium based compounds. Nanoparticles size *versus* different C_{CAs}: (Gd-DTPA (- ■ -); $GdCl_3$ (- ● -)); at *standard conditions* showing how the stability of the hydrodynamic flow-focusing is compromised when Gd-DTPA is added to the solvent phase. No interference is observed in presence of $GdCl_3$at different concentration.

The stability of the hydrodynamic flow-focusing results altered when Gd-DTPA is added to the solutions (Figure S5a and b) and, consequently, the morphology of the nanoparticles is compromised (Figure S5c and d).

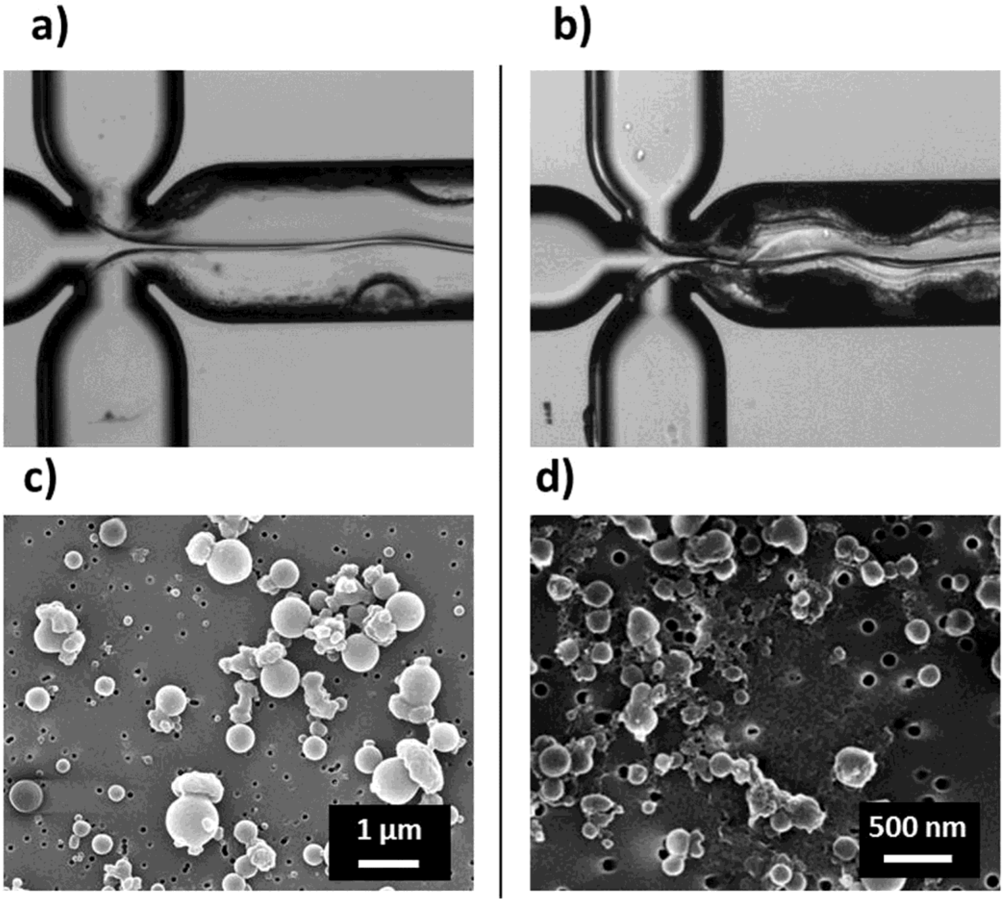

Figure S5. Influence of Gd-DTPA on the nanoprecipitation. Instability of the Hydrodynamic Flow-Focusing when Gd-DTPA is added to the solvent phase. a) at the beginning and b) at the end of the experiment. FE-SEM images of larger and aggregated morphologies obtained at the concentration of Gd-DTPA of c) 0.1% wt/v, d) 0.05% wt/v.

Effect of NaCl

Typically, a change in NaCl results in an increase of the crosslinking degree of the hydrogel nanoparticles. However, Figure S6a shows how a further increase of NaCl concentration results in a massive and uncontrolled precipitation. The optimal value of NaCl concentration to obtain nanoparticle morphologies is 0.05 M; indeed, at higher concentrations, it is only possible to observe fiber-like morphologies mixed to nanoparticles (Figure S6b).

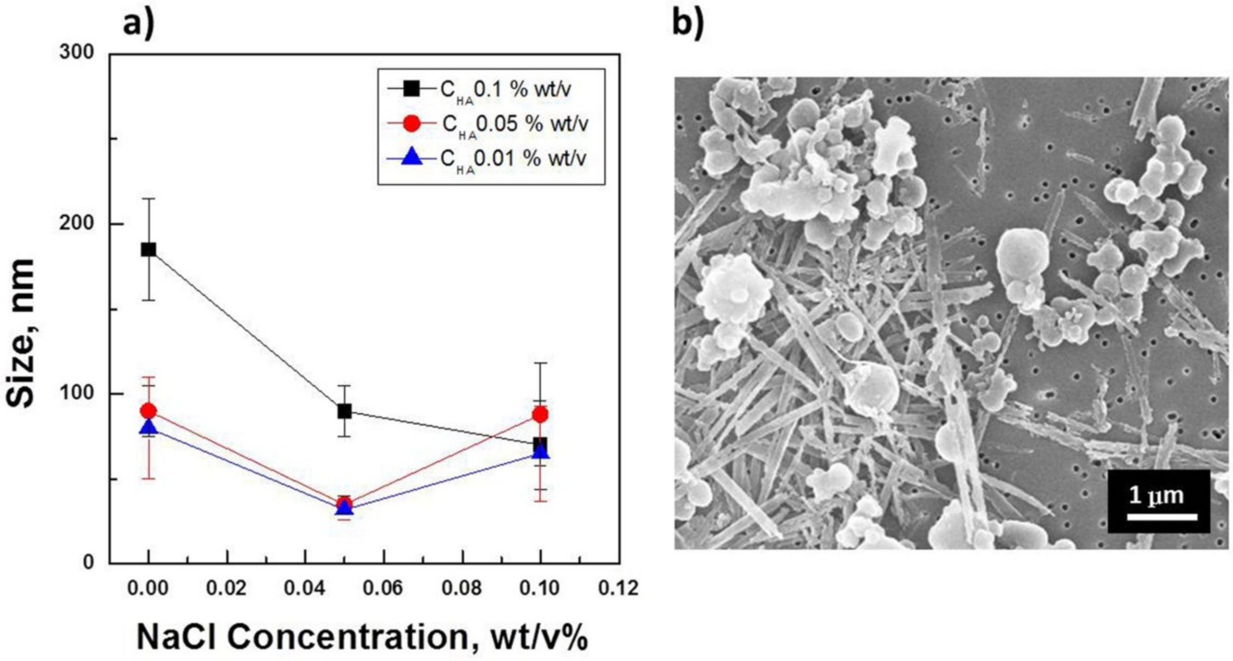

Figure S6. Influence of NaCl on nanoparticles morphologies. a) Nanoparticles size *versus* NaCl concentration by varying C_{HA} (0.01 % wt/v (- ▲ -); 0.05 % wt/v (- ● -); 0.1 % wt/v (- ■ -); b) FE-SEM image of morphologies at 0.1M NaCl concentration showing fiber-like morphologies.

Loading capability and Encapsulation efficiency of Gd-DTPA in cHANPs

The lyophilization of cHANPs was performed using a freeze-dryer Christ, 1-4LSC. Briefly, a freezing step was conducted for 3 hr at 80 °C. A cooling profile of 1 °C/min was applied, sublimation at a shelf temperature of 6 °C and pressure of 0.85 mbar for at least 24 hr[2]. Finally, a secondary drying at 25 °C and 0.03 mbar was performed for 5-6 hr. Trehalose or sucrose ranging

from 1-3% wt/v was added as cryoprotector if necessary. Dried particles were also observed by Field Emission-SEM.

The quantification of drug loading and the encapsulation efficiency was determined weighing a certain amount of particles, typically 1 mg, into each of three 1.5 mL ultracentrifuge tubes. Dry particles were collected at the bottom of the tube by performing a quick spin-down (80.000 rpm – 45 minutes – 15°C). Two methods were utilized to solubilize the compound contained in the particles: dissolving the particles in a solvent or swelling the particles to allow the release of the complex into solution by shaking at 37°C for 1hr. The equations used to determine the loading capability $\boldsymbol{LC}$ and the encapsulation efficiency $\boldsymbol{EE}$ are[3]:

$$\%\ \boldsymbol{LC} = \boldsymbol{C_i V_i / m_{np_s}}$$

$$\%\ \boldsymbol{EE} = \left(\boldsymbol{C_i V_i / m_{np_s}}\right) / \left(\boldsymbol{m_{Gd}} / \left(\boldsymbol{m_{Gd}} + \boldsymbol{m_{HA}}\right)\right)$$

Strategies to produce cHANPs by controlling the flow-focusing pattern in presence of Gd-DTPA

The addition of the Gd-DTPA results in larger and high polydisperse nanoparticles. To control this interference and to restore optimized process parameters, we explore two different strategies: the tuning of pH conditions and the addition of hydro or lipophilic surfactants. In the first case, pH conditions are accurately tuned in the HA solution by varying NaOH concentration and keeping constant NaCl to avoid massive precipitation (Figure S6). To restore the previous morphology, a narrow range of pH values, from 11.5 to 12.5, is found to be appropriate to control the interference between Gd-DTPA and HA matrix. Indeed, the variation of pH in the middle channel, when Gd-DTPA is also added, strongly influences the flow-focusing pattern by reducing the flow perturbations within the microfluidic device. Nanoparticles of around 40 nm in diameter are obtained for this range of pH. Furthermore, the significant success of this strategy can not only be found in the reduced size of the nanoparticles but also into the considerable increase of the Gd-DTPA concentration in the HA solution from 0.005 to 0.1% wt/v. Figure S7a clearly shows the effect of pH on the nanoparticle size due to the improved stability of the flow-focusing. This first strategy confirms that the addition of alkali changes the conformation of the molecule through the breaking of hydrogen bonds, therefore influencing the interference with the Gd-DTPA, and the polymer molecules begin to untangle from each other, aligning in the direction of flow and resulting in a drastic reduction in viscosity. This optimal pH range reduces the viscosity of the medium and anomalous precipitations and promotes the degree of crosslinking of the NPs, as reported in the next paragraphs. The second proposed strategy is exploited by adding surfactants to the side channels to support the streams during the focusing action and to modulate the nanoprecipitation. The optimization of the flow-focusing pattern is found to be strongly dependent on the hydrophobicity and hydrophilicity

of the surfactants. Indeed, the good hydrophilicity of the Tween 21 (HLB =13.3) highly interferes with the extraction of the water solvent by increasing instability of the focusing and, consequently, inducing agglomeration or polydispersity of the observed sample. Contrarily, when the less hydrophilic Tween85 (HLB=11) is added to the non-solvent, the flow-focusing appears very stable, compared to the previous one, because the lower compatibility of the Tween85 with the water solvent reduces the formation of heterogeneities at the interface. However, for both surfactants, a slight increase of the nanoparticle size is observed (Figure S7b). Following the previous results, a more hydrophobic surfactant is tested. Span80 (HLB=4.3) is highly insoluble in water but presents good solvation in alcohol. Indeed, when Span80 is added to the non-solvent, stability of the focusing strongly increases in comparison to the other surfactants. This significant result shows that it is possible to reduce the interferences at the solvent-non-solvent interface using an appropriate surfactant that can simply modulate the affinity between solvent and non-solvent, regaining the control of the flow-focusing pattern and, consequently, the morphology of the nanoparticles (Figure S7c and d). For completeness of results, a few tests are also performed by adding the water-soluble surfactants to the middle channel, but the formation of nanostructured morphologies is not observed. These strategies are also conducted at different pH values (Table S2), confirming the preferred range of pH even in the presence of surfactants.

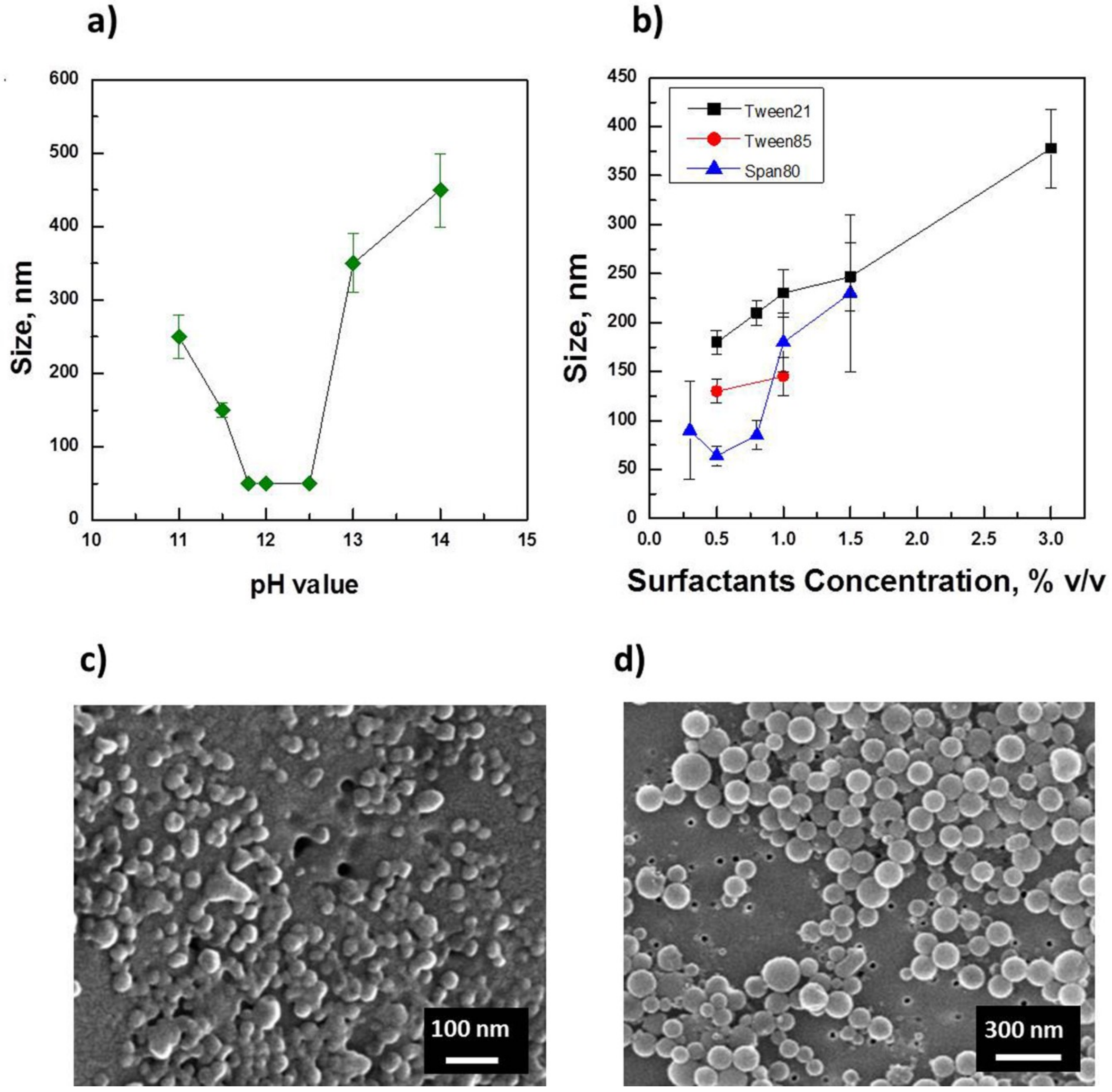

Figure S7. Strategies to control the interference of Gd-DTPA on Flow-Focusing. Size of nanoparticles at C_{HA} 0.05% wt/v and at Gd-DTPA 0.1% wt/v reported for several a) pH values, b) concentration of surfactants; FE-SEM images of nanoparticles obtained at *standard conditions*: c) tilted image at 15° after the optimization of the pH value, d) not tilted image in presence of the hydrophilic surfactant Tween21 at 0.5% v/v. Data are collected without performing the crosslinking reaction and nanoparticles size is observed in Ethanol.

Table S2. Optimization of loading capability and crosslinking reaction. Process conditions of the investigated strategies to control the interference of Gd-DTPA in the nanoprecipitation mechanism: concentration of surfactants and tuning of pH condition together with the concentration of DVS added to the middle channel and to the side channel.

**SURFACTANTS**	**Ph**	
$0.5\ \%\ v/v < Tween21 < 3\ \%\ v/v$<br>$0.5\ \%\ v/v < Tween85 < 1.5\ \%\ v/v$<br>$0.5\ \%\ v/v < Span80 < 1.5\ \%\ v/v$	$11 < pH < 14$	**DVS**<br>**side channels**<br>$0.4\ \%\ v/v < CDVS < 5\%\ v/v$
$0.5\ \%\ v/v < Span80 < 1.5\ \%\ v/v$	$11 < pH < 14$	**DVS**<br>**middle channel**<br>$0.4\ \%\ v/v < CDVS < 6\%\ v/v$<br>Inlet channel $T \cong 5°C$<br>Mixing channel $T \cong 35°C$

General observation about the interference of Gd-based CAs on Nucleation and Growth in Microfluidics

In the traditional nanoprecipitation, the mixing time drives the formation of nanoparticles of a certain size (Figure S8a). In the flow-focused nanoprecipitation, when a stable and thin hydrodynamic flow-focusing is achieved along the main channel, the mixing time can be extremely reduced, and a fast extraction occurs at the nozzle section by providing a high level of mixing for all species (Figure S8b). In our microfluidic system, a high molecular weight polymer solution is added to the middle channel and focused by the non-solvent lateral flows. In these conditions, a rapid increase in the concentration of free polymer chains into the solution takes place, inducing a "burst nucleation" which makes other free polymer chains in solution unavailable. Indeed, during this "effective nucleation", an increase in the rate of nucleation into the main channel produces a strong dilution of the polymer chains that limits the diffusion of the chains through the solution; consequently, reducing the growth by diffusion (Figure S8b). However, the stability and the width of the hydrodynamic flow-focusing are altered when even small concentrations of Gd-DTPA are added to the solutions (Figure S8c), compromising morphology, size and loading capability of the nanoparticles.

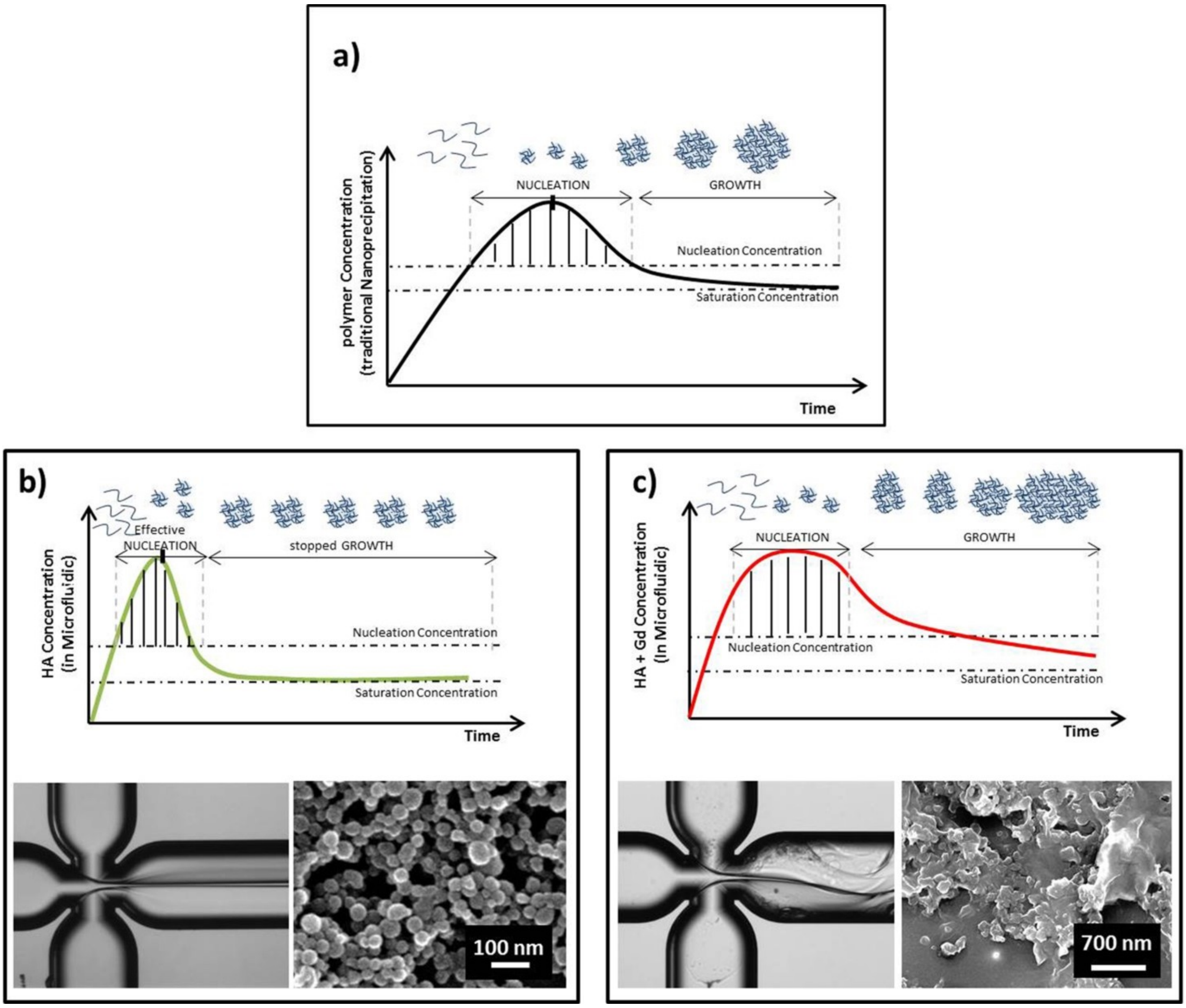

Figure S8. Interference of contrast agent on the Nanoprecipitation mechanism. The scheme reports the interpretation of the nucleation and growth phenomena a) in a traditional batch system; b) in a microfluidic device at *standard conditions*; c) in a microfluidic device when Gd-DTPA is added to the solvent phase at standard conditions. Panel a) shows the well-known thermodynamics related to the batch processes reported in the table for different polymers-solvent-nonsolvent systems. Panel b) represents the interpretation of the thermodynamics when nanoprecipitation occurs in a microfluidic device and a stable focused stream produces monodisperse nanoparticle by an *effective nucleation.* Panel c) represents changes of the nanoprecipitation phases, when Gd-DTPA is added to the solvent, and instability of the flow-focusing produces aggregated and polydisperse nanoparticles from a slow heterogeneous nucleation.

References

1. L. Capretto, W. Cheng, M. Hill and X. Zhang, *Microfluidics: Technologies and Applications*, 2011, **304**, 27-68.
2. W. Abdelwahed, G. Degobert, S. Stainmesse and H. Fessi, *Advanced Drug Delivery Reviews*, 2006, **58**, 1688-1713.
3. J. A. Ankrum, O. R. Miranda, K. S. Ng, D. Sarkar, C. Xu and J. M. Karp, *Nature Protocols*, 2014, **9**, 233-245.
